# 免疫抑制剂FK506对肺癌细胞增殖和迁移的作用研究

**DOI:** 10.3779/j.issn.1009-3419.2017.07.02

**Published:** 2017-07-20

**Authors:** 永文 李, 洪兵 张, 颖 李, 辰龙 赵, 伟婷 李, 红雨 刘, 建平 闻, 军 陈

**Affiliations:** 1 300072 天津，天津大学化工学院生物工程系 Department of Biological Engineering, School of Chemical Engineering and Technology, Tianjin University, Tianjin 300072, China; 2 300052 天津，天津医科大学总医院肺癌研究所 Tianjin Lung Cancer Institute, Tianjin Medical University General Hospital, Tianjin 300052, China; 3 300052 天津，天津医科大学总医院肺部肿瘤外科 Department of Lung Cancer Surgery, Tianjin Medical University General Hospital, Tianjin 300052, China

**Keywords:** 肺肿瘤, FK506, 抗肿瘤作用, Lung neoplasms, FK506, Antitumor effect

## Abstract

**背景与目的:**

FK506（他克莫司，tacrolimus）是一种大环内酯类的新型免疫抑制剂。研究报道FK506对多种肿瘤细胞具有增殖抑制作用。本研究旨在观察FK506对肺癌细胞增殖和迁移的作用，并探讨其可能的机制。

**方法:**

体外培养A549和H1299细胞，采用CCK-8法测定FK506对A549和H1299细胞增殖的作用；EDU标记法检测DNA合成；流式细胞术检测细胞的周期分布情况；Transwell和细胞划痕实验检测细胞的体外迁移能力；Western blot技术检测P27、RB1、CDK4、CDK6和MMP9蛋白的表达。

**结果:**

FK506可抑制A549和H1299细胞的增殖、诱导细胞周期G_0_期/G_1_期阻滞；与对照组比较，FK506处理后A549和H1299细胞迁移能力明显降低，且呈剂量依赖性。此外，与对照组相比，FK506处理组中P27和RB1表达上调，而CDK4、CDK6和MMP9表达显著下降。

**结论:**

FK506对人肺癌A549和H1299细胞的增殖和迁移能力有明显的抑制作用，其机制可能与上调p27表达、抑制CDK4、CDK6和MMP-9表达有关。

## Abstract (en)

**Background and objective:**

FK506, also named tacrolimus, a new macrolide immunosuppressive agent, has been shown to possess anti-proliferation activities in some cancer cells. The aim of this study was to investigate the effect of FK506 on the cell proliferation and migration of lung cancer cell lines and its mechanism.

**Methods:**

A549 and H1299 cell lines were cultured *in vitro*. The effect of FK506 on cell viability and DNA synthesis ability of A549 and H1299 were measured by CCK-8 assay and EDU-labeling assay, respectively. Flow cytometry assay was used to detect the cell cycle. The *in vitro* migration of lung cancer cells was detected by Boyden chamber assay and wound-healing assay after the treatment of FK506. The expression of p27, RB1, CDK4, CDK6 and MMP9 were detected using Western blot.

**Results:**

FK506 inhibited cell growth and induced cell cycle arrest in G_0_/G_1_ phase in A549 and H1299 cells in a dose-and time-dependent manner. Compared to the control groups, the migration of A549 and H1299 cells treated with FK506 were decreased obviously. Moreover, FK506 increased the expression of P27 and RB1, and reduced the expression of CDK4, CDK6 and MMP9.

**Conclusion:**

FK506 inhibit the cell growth and migration of lung cancer cells *in vitro*. The inhibitive effects may be associated with the up-regulation of p27 expression and inhibition CDK4, CDK6 and MMP9 expression.

肺癌是最常见的恶性肿瘤之一，也是发病率和死亡率增长最快、对人类健康和生命威胁最大的恶性肿瘤之一。肺癌具有高发病率、易复发、难治疗的特点，5年生存率仅为20%-30%^[1, 2]^。临床治疗多采用手术切除，并联合放疗或化疗以及靶向治疗^[3, 4]^。但由于癌细胞的高侵袭性和迁移性，易发生复发和转移。因此，寻找肿瘤细胞的新的抑制剂成为抗肺癌研究和治疗中的重要策略。

新型免疫抑制剂在临床上广泛应用，不仅在防止器官移植免疫排斥的治疗上发挥重要作用，极大改善了器官移植患者的生存质量，同时也为许多自身免疫性疾病的治疗开辟了广阔的应用前景^[5]^。FK506（tacrolimus，他克莫司）是从链霉菌属（Streptomyces）中分离出的23元大环内酯类新型免疫抑制剂^[6, 7]^。近年来，肝移植术、肾移植术已日益成熟，并广泛用于治疗严重肝脏、肾脏疾病。而肺移植也逐渐成为治疗严重慢性阻塞性肺疾病（chronic obstructive pulmonary disease, COPD）、特发性肺纤维化等肺部疾病的重要手段，并为肺癌患者带来希望。而移植术后肿瘤的发生和转移复发也引起了人们格外的关注。有研究^[8-10]^报道，肝癌肝移植术后免疫抑制剂的应用可能促进了术后肿瘤的转移复发。而免疫抑制剂环孢素A（cyclosporin A, CsA）和FK506对膀胱癌、前列腺癌、泌尿道肿瘤等细胞具有明显的抗肿瘤作用，但关于肺癌与FK506药物的相关研究罕见报道。此外，FK506药物抗肿瘤作用的分子机制仍不清楚。本研究的目的是研究FK506对人肺癌细胞株A549和H1299的抗肿瘤作用，并对其相关机制进行初步探讨。

## 1 材料和方法

### 1.1 材料

细胞株：人肺癌细胞株A549和H1299购自中国科学院典型培养物保藏委员会细胞库，用含10%胎牛血清的RPMI-1640培养基于37 ℃、5%CO_2_饱和湿度条件的培养箱内进行常规传代培养。试剂：免疫抑制剂FK506（纯度≥98%，天津苏斯泰来生物技术有限公司闻建华老师提供）；RPMI-1640培养基和胎牛血清（Gibco）；Trizol（Life Technology）；CCK-8试剂盒（碧云天）；Cell-Light^TM^ EdU检测试剂盒（广州市锐博生物科技有限公司）；细胞周期分析试剂盒（BD Biosciences）；BCA蛋白浓度测定试剂盒（Thermo Fisher Scientific）；鼠抗人p27、细胞周期蛋白依赖性激酶（cyclin-dependent kinase, CDK）4、CDK6、RB1（Santa Cruz Biotechnology）以及MMP9单克隆抗体（Cell Signaling Technology）；β-actin单克隆抗体（Sigma-Aldrich）；抗鼠辣根过氧化物酶标记Ⅱ抗（Thermo Fisher Scientific）；增强化学发光（ECL）试剂（Thermo Fisher Scientific）；Transwell小室和Matrigel（Corning）。

### 1.2 CCK-8法检测FK506对细胞增殖的影响

处于对数生长期的A549和H1299细胞铺于96孔板中，用不同浓度的FK506（10 μmol/L、20 μmol/L、40 μmol/L、60 μmol/L、80 μmol/L和100 μmol/L）处理细胞24 h和48 h后，每个药物浓度每个时点5个复孔，每孔加入10 μL CCK-8试剂于37 ℃中继续培养2 h，然后于酶标仪上测定450 nm的吸光度A。细胞增殖抑制率（%）=（1-实验组平均A值/正常对照组平均A值）×100%。细胞的相对活性（%）=1-细胞增殖抑制率。

### 1.3 EdU标记实验

将A549和H1299细胞分别以4×103接种于96孔板中，在0、30 μmol/L和60 μmol/L FK506处理细胞48 h后，每孔加入100 μL含EDU培养基孵育2 h，经4%多聚甲醛和0.5% Triton X-100处理后，加入100 μL的Apollo染色反应液，并用含DAPI的荧光封片剂进行封片，荧光显微镜下观察检测。

### 1.4 细胞周期分析

取对数生长期的A549和H1299细胞，2×10^5^/mL接种于10 cm培养皿，30 μmol/L和60 μmol/L FK506碱处理48 h后，用0.25%的胰酶消化，2, 000 r/min离心5 min收集细胞，PBS洗2次，1, 000 r/min离心后弃上清液，500 μL 70%乙醇4 ℃固定过夜。加入RNA酶消化，使用碘化丙啶（PI）避光染色30 min，BD流式细胞仪进行细胞周期分析，重复实验3次。

### 1.5 Transwell细胞迁移实验

Transwell小室中加入100 μL的含1×10^5^细胞悬液，下室加入600 μL完全培养基，置于37 ℃ 5%CO_2_恒温孵箱培养24 h。取出Transwell小室用棉签小心拭去基质胶及上室细胞，4%多聚甲醛固定10 min，结晶紫室温染色25 min，PBS洗3遍，显微镜下选5个随机视野进行拍照计数。计算平均每个视野的细胞数，以穿过滤膜进入下室的细胞数来表示细胞的侵袭能力。每组实验重复3次。

### 1.6 细胞划痕实验

A549和H1299细胞接种于6孔板中，形成单层贴壁细胞后用FK506处理，当细胞长至95%以上融合度后，用200 μL的移液器枪头在单层细胞上划痕，用PBS洗去散落的细胞，加入含0.1%FBS的RPMI-1640培养基，37 ℃、5%CO_2_细胞培养箱继续培养24 h。按0、24 h取样拍照。迁移程度=（0 h两侧距离-24 h两侧距离）/0 h两侧距离。

### 1.7 免疫印迹实验

取对数生长期的细胞，FK506（0、30 μmol/L和60 μmol/L）分别作用于细胞48 h后，冰上收集细胞，PBS洗涤2次后离心，加入RIPA裂解30 min，并低温离心，收集上清并用BCA法测定蛋白浓度。经10%SDS-PAGE电泳分离后电转印至硝酸纤维素膜（nitrocellulose filter membrane，简称NC膜）上。5%脱脂牛奶封闭2 h后，加入p27（1:200）、RB1（1:1, 000）、CDK4（1:200）、CDK6（1:1, 000）以及MMP9（1:1, 000）一抗4 ℃孵育过夜；TBST洗涤3次×5 min；加入1:2, 000稀释的HRP标记的二抗，室温孵育1 h，TBST洗膜3次×5 min，增强化学发光法显影，应用凝胶成像分析系统分析结果。

### 1.8 统计学方法

采用SPSS 21.0统计软件包进行分析。计量资料以均数±标准差（Mean±SD）表示，各组之间的比较采用*ANOVA*检验。*P*＜0.05为差异有统计学意义。

## 2 结果

### 2.1 FK506对肺癌细胞增殖的抑制作用

CCK-8实验结果显示，细胞经不同浓度的FK506处理24 h和48 h后，A549和H1299细胞增殖明显受到抑制（图 1），与对照组相比，FK506处理组中细胞的抑制率明显上调，且其作用效果呈浓度和时间依赖性。FK506处理24 h对A549和H1299细胞生长抑制的IC_50_分别为38.14 μmol/L和39.09 μmol/L，而处理48 h后A549和H1299细胞的IC_50_分别为34.98 μmol/L和23.40 μmol/L，后续实验主要选用30 μmol/L和60 μmol/L FK506处理细胞。

**1:**
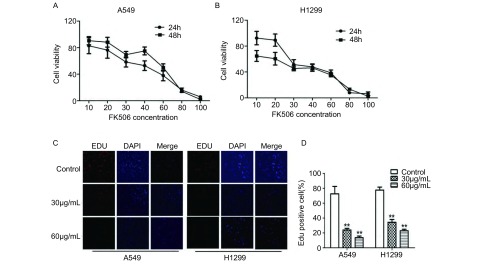
FK506对肺癌肿瘤细胞株A549和H1299生长的抑制作用。A：CCK-8试验结果显示FK506明显抑制A549细胞的活性；B：CCK-8试验结果显示FK506明显抑制H1299细胞的活性；C和D：EDU标记试验检测FK506对A549和H1299细胞DNA合成能力的影响。^**^P＜0.01。 Inhibitory effect of FK506 on the growth of A549 and H1299 lung cancer cells. A: FK506 inhibited the cell viability of A549 cells using CCK-8 assay; B: FK506 inhibited the cell viability of H1299 cells using CCK-8 assay; C and D: FK506 inhibited DNA synthesis ability of A549 and H1299 cells using EDU-labeling assay. ^**^P < 0.01.

另外，EdU标记实验结果显示，相比于对照组，30 μmol/L和60 μmol/L FK506处理组中A549细胞和H1299细胞EDU阳性率明显下降（*P*＜0.01），且随着药物浓度的增加而呈下降趋势。

### 2.2 FK506对肺癌细胞周期分布的影响

分别用30 μmol/L和60 μmol/L FK506处理A549和H1299细胞，然后采用了流式细胞术检测FK506处理后的细胞周期分布情况，结果显示FK506处理48 h后，A549和H1299细胞中G_0_/G_1_细胞的比例明显高于对照组，而G_2_期/M期的细胞的比例明显低于对照组，且呈药物浓度依赖性，提示FK506能诱导肺癌细胞的G_0_期/G_1_期细胞停滞（图 2）。

**2:**
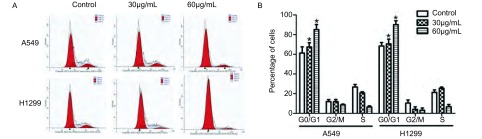
FK506对A549和H1299细胞周期分布的影响。A：流式细胞技术检测FK506处理细胞48 h后细胞周期分布；B：细胞周期分布直方图。^*^P＜0.05。 Effects of FK506 on cell cycle distribution of A549 and H1299 cells. A: Cell cycle distribution was detected by flow cytometry using a propidium iodide staining assay after treatment with FK506 for 48 h; B: Histogram of cell cycle distribution. ^*^P < 0.05.

### 2.3 FK506抑制肺癌细胞迁移能力

分别通过Transwell法及划痕实验检测细胞的迁移能力，FK506处理细胞48 h后，与对照组相比较，A549和H1299细胞FK506处理组中穿过膜的细胞数量明显减少（图 3）。此外，细胞划痕实验结果显示，FK506处理24 h和48 h后，30 μmol/L和60 μmol/L FK506处理组的A549和H1299细胞的迁移率与对照组相比细胞迁移率明显下降（*P*＜0.05），而且随着药物浓度的增加，细胞迁移率有下降趋势，差异具有统计学意义（*P*＜0.05）（图 4）。

**3:**
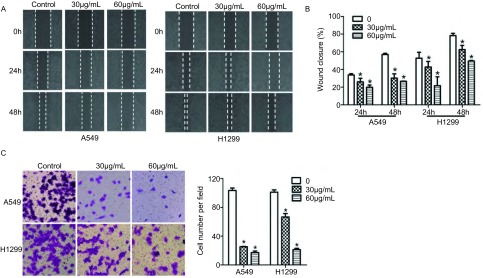
FK506对A549和H1299细胞迁移的影响。A：细胞划痕实验检测FK506细胞处理48 h后细胞迁移能力；B：细胞迁移能力统计直方图；C：Transwell细胞迁移实验检测FK506处理细胞48 h后细胞迁移能力。^*^P＜0.05。 Effects of FK506 on cell migration of A549 and H1299 cells. A: Cell migration was detected by wound-healing assay after treatment with FK506 for 48 h; B: Histogram of cell migration of A549 and H1299 cells; C: Transwell migration assay was used to detect the effect of FK506 on cell migration after treatment with FK506 for 48 h. ^*^P < 0.05.

**4:**
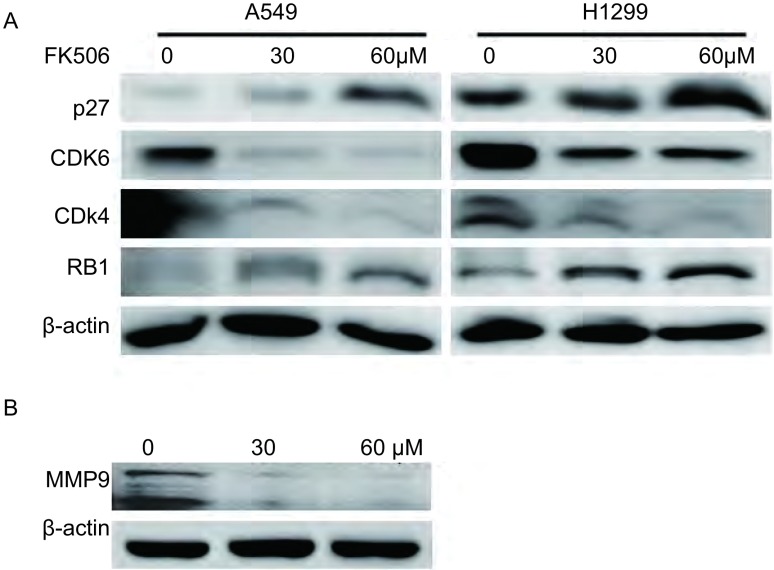
FK506对细胞周期相关因子和MMP9表达的影响。A：Western blot检测细胞周期相关因子的表达；B：Western blot检测MMP9的表达。 Effects of FK506 on expression of cell cycle-related proteins and MMP9 expression. A: The expression of cell cycle-related proteins were detected by Western blot; B: The expression of MMP9 was detected by Western blot.

### 2.4 FK506调控细胞周期相关因子和MMP9的表达

为进一步探索FK506抑制肺癌细胞增殖和迁移能力的分子机制，我们还采用了免疫印迹技术检测细胞周期相关调控蛋白p27、RB1、CDK4和CDK6的表达。结果发现，FK506能显著抑制人肺癌细胞A549和H1299的CDK4、CDK6和RB1的蛋白表达，而升高p27的表达水平。另外，同对照组相比，FK506处理后A549细胞中MMP9的表达显著下调。

## 3 讨论

近年来我国肺癌的发病率呈明显上升趋势，尽管近年来临床治疗取得了很大进展，但死亡率仍然很高，5年生存率仅为20%-30%^[1]^。因此，研究肺癌新的治疗药物具有重要的意义。FK506不仅在器官移植抗免疫排斥中发挥重要作用，还对严重的、顽固性的狼疮性皮肤损害有良好效果。近年来研究还表明，FK506能抑制肿瘤细胞的增殖，但其在肺癌细胞的相关作用尚未见报道。本研究以A549和H1299细胞株为研究对象，探讨FK506在体外对肺癌细胞株的增殖和细胞迁移的作用及相关机制。

本研究证实了FK506对肺癌细胞株A549和H1299有明显的生长抑制作用，且对肺癌细胞的抑制作用呈时间和剂量依赖性。细胞周期调控的异常与肿瘤的发生及发展密切相关。我们检测了FK506处理对肺癌细胞周期分布的影响，发现FK506处理能诱导肺癌细胞停滞在G_0_期-G_1_期增多，且呈剂量依赖性。*P27*基因是近年来发现的一个重要的抑癌基因，其编码的P27蛋白是细胞周期调控中重要的控制因子^[11]^。P27主要通过抑制cyclin和CDK2/4等G_1_期激酶复合物的活性，从而阻止细胞由G_1_期向S期的转变^[12]^。CDK4和CDK6是蛋白激酶家族的重要成员，通过与cyclin形成复合物磷酸化一系列的目标底物，从而导致细胞周期循环的产生。CDK4和CDK6水平的下降也可通过阻止转录因子E2F的释放，从而抑制DNA的合成^[13]^。抑癌基因*RB1*在细胞周期G_0_期、G_1_期处于非磷酸化状态，并与转录因子E2F结合，从而抑制靶基因活化。本课题组研究发现，FK506能显著抑制P27的表达水平，抑制周期蛋白依赖性激酶CDK4和CDK6的表达水平，提示FK506可能通过上调P27的表达水平，从而抑制CDK4和CDK6的水平，进而抑制DNA的合成。由此可推断，诱导细胞周期停滞可能是FK506抑制肺癌细胞增殖的途径之一。

肿瘤的转移是一复杂的多步骤过程，寻找新的能有效抑制恶性肿瘤的复发、侵袭及迁移的肿瘤抑制剂，是抗癌药物研究的方向。本研究证实了FK506能抑制肺癌细胞的体外迁移。基质金属蛋白酶（matrix metalloproteinases, MMPs）是一类分解细胞外基质组分的锌蛋白酶，主要的生理作用是降解细胞外基质（extracellular matrix, ECM），破坏肿瘤细胞侵袭的组织学屏障^[14]^。MMPs已被证明在肿瘤生长、侵袭和转移中发挥着关键作用。MMP9是MMPs中研究较深入的明胶酶，主要水解变性胶原及基膜的主要成分Ⅳ型胶原，与肿瘤的侵袭和转移密切相关^[15, 16]^。本研究结果表明，FK506对MMP9的抑制作用随着药物浓度的增强而增强，提示FK506对肺癌细胞侵袭和迁移能力的抑制与MMP9活力下降相关。

综上所述，通过体外实验我们初步证实FK506可以抑制肺癌A549和H1299细胞的增殖和迁移能力。FK506可能通过诱导细胞周期G_0_/G_1_停滞而抑制肺癌细胞的增殖，并且其对肺癌细胞迁移能力的抑制可能与MMP9活力下降相关。

## References

[1] Minna JD, Roth JA, Gazdar AF (2002). Focus on lung cancer. Cancer Cell.

[2] Jemal A, Bray F, Center MM (2011). Global cancer statistics. CA Cancer J Clin.

[3] Gettinger S (2008). Targeted therapy in advanced non-small-cell lung cancer. Semin Respir Crit Care Med.

[4] Rosell R, Karachaliou N (2015). Lung cancer in 2014: optimizing lung cancer treatment approaches. Nat Rev Clin Oncol.

[5] Ziaei S, Halaby R (2016). Immunosuppressive, anti-inflammatory and anti-cancer properties of triptolide: a mini review. Avicenna J Phytomed.

[6] Kahan BD (2003). Timeline: individuality: the barrier to optimal immuno-suppression. Nat Rev Immunol.

[7] Haufroid V, Mourad M, Van Kerckhove V (2004). The effect of *CYP3A*5 and *MDR1* (*ABCB1*) polymorphisms on cyclosporine and tacrolimus dose requirements and trough blood levels in stable renal transplant patients. Pharmacogenetics.

[8] Kawahara T, Kashiwagi E, Ide H (2015). The role of NFATc1 in prostate cancer progression: cyclosporine A and tacrolimus inhibit cell proliferation, migration, and invasion. Prostate.

[9] Kawahara T, Kashiwagi E, Ide H (2015). Cyclosporine A and tacrolimus inhibit bladder cancer growth through down-regulation of NFATc1. Oncotarget.

[10] Kawahara T, Kashiwagi E, Li Y (2016). Cyclosporine A and tacrolimus inhibit urothelial tumorigenesis. Mol Carcinog.

[11] Polyak K, Lee MH, Erdjument-Bromage H (1994). Cloning of p27Kip1, a cyclin-dependent kinase inhibitor and a potential mediator of extracellular antimitogenic signals. Cell.

[12] Sotillo R, Renner O, Dubus P (2005). Cooperation between Cdk4 and p27kip1 in tumor development: a preclinical model to evaluate cell cycle inhibitors with therapeutic activity. Cancer Res.

[13] Paternot S, Bockstaele L, Bisteau X (2010). Rb inactivation in cell cycle and cancer: the puzzle of highly regulated activating phosphorylation of CDK4 versus constitutively active CDK-activating kinase. Cell Cycle.

[14] Verma RP, Hansch C (2007). Matrix metalloproteinases (MMPs): chemical-biological functions and (Q)SARs. Bioorg Med Chem.

[15] Nagase H, Woessner JF Jr (1999). Matrix metalloproteinases. J Biol Chem.

[16] Groblewska M, Siewko M, Mroczko B (2012). The role of matrix metalloproteinases (MMPs) and their inhibitors (TIMPs) in the development of esophageal cancer. Folia Histochem Cytobiol.

